# Multidrug-resistant tuberculosis treatment adherence in migrants: a systematic review and meta-analysis

**DOI:** 10.1186/s12916-017-1001-7

**Published:** 2018-02-22

**Authors:** Laura B. Nellums, Kieran Rustage, Sally Hargreaves, Jon S. Friedland

**Affiliations:** Infectious Diseases & Immunity, Department of Medicine, Imperial College London, Hammersmith Hospital, Du Cane Road, London, W12 ONN UK

**Keywords:** Tuberculosis, Drug resistance, Migration, Treatment adherence

## Abstract

**Background:**

Multidrug-resistant tuberculosis (MDR-TB) is a growing concern in meeting global targets for TB control. In high-income low-TB-incidence countries, a disproportionate number of MDR-TB cases occur in migrant (foreign-born) populations, with concerns about low adherence rates in these patients compared to the host non-migrant population. Tackling MDR-TB in this context may, therefore, require unique approaches. We conducted a systematic review and meta-analysis to identify and synthesise data on MDR-TB treatment adherence in migrant patients to inform evidence-based strategies to improve care pathways and health outcomes in this group.

**Methods:**

This systematic review and meta-analysis was conducted in line with PRISMA guidelines (PROSPERO 42017070756). The databases Embase, MEDLINE, Global Health and PubMed were searched to 24 May 2017 for primary research reporting MDR-TB treatment adherence and outcomes in migrant populations, with no restrictions on dates or language. A meta-analysis was conducted using random-effects models.

**Results:**

From 413 papers identified in the database search, 15 studies reporting on MDR-TB treatment outcomes for 258 migrants and 174 non-migrants were included in the systematic review and meta-analysis. The estimated rate of adherence to MDR-TB treatment across migrant patients was 71% [95% confidence interval (CI) = 58–84%], with non-adherence reported among 20% (95% CI = 4–37%) of migrant patients. A key finding was that there were no differences in estimated rates of adherence [risk ratio (RR) = 1.05; 95% CI = 0.82–1.34] or non-adherence (RR = 0.97; 95% CI = 0.79–1.36) between migrants and non-migrants.

**Conclusions:**

MDR-TB treatment adherence rates among migrants in high-income low-TB-incidence countries are approaching global targets for treatment success (75%), and are comparable to rates in non-migrants. The findings highlight that only just over 70% of migrant and non-migrant patients adhere to MDR-TB treatment. The results point to the importance of increasing adherence in all patient groups, including migrants, with an emphasis on tailoring care based on social risk factors for poor adherence. We believe that MDR-TB treatment targets are not ambitious enough.

## Background

Multidrug-resistant tuberculosis (MDR-TB), defined by resistance to isoniazid and rifampicin, the two front-line antimicrobial drugs used to treat TB, presents substantial barriers to the eradication of global TB due to challenges around diagnosis and successful treatment [1–4]. As a result, MDR-TB has become a major focus for TB research in recent years [1, 2], with 480,000 new MDR-TB cases recorded in 2015 [1], comprising 5% of the total global TB burden [5]. Current evidence suggests that MDR-TB will increase globally as a proportion of total TB cases, not only due to transmission of MDR-TB [6, 7], but also poor adherence to TB treatment leading to the emergence of MDR-TB [1, 8–11]. World Health Organization (WHO) global targets for MDR-TB treatment success and adherence are currently 75%, which is lower than the 85% target for drug-sensitive TB [1, 12], reflecting both the higher rates of mortality and barriers to treatment adherence.

It is estimated that there are over 244 million international migrants worldwide [13], with rates of migration increasing, which has significant implications for global health and health systems internationally [14, 15]. In many high-income low-TB-incidence countries, migrant (foreign-born) populations experience a disproportionate burden of TB and MDR-TB [16–18]. Furthermore, they are suggested to have poorer treatment outcomes, with 5.2% of non-UK-born TB cases notified as being lost to follow-up, compared to only 1.5% among UK-born TB cases [19]. As a result, they have received particularly close attention in targeted TB initiatives [20, 21], with specific frameworks now being drawn up globally to tackle TB and MDR-TB within these communities [22].

MDR-TB treatment regimens may be of long duration. Whilst a shorter 9-month Bangladesh treatment regimen is available for some patients, in many cases MDR-TB treatment requires an intensive treatment phase up to 8 months in duration, with a further 12-month minimum continuation phase [23]. Treatment for MDR-TB typically utilises at least five active drugs depending on the specific resistance profile of the infection and includes second-line drugs, which have increased toxicity, worse side effects, greater treatment burden (e.g. number of pills) and reduced individual efficacy [1, 21, 23–27]. As a result of these factors, adherence rates are typically lower than for drug-sensitive TB, though the consequences of non-adherence are more severe. There are currently limited data on MDR-TB treatment adherence in migrant patients, and a lack of clarity in health initiatives globally as to the priorities for improving care – and ultimately treatment outcomes – in these populations [1, 28]. Though there is an insufficient evidence base regarding treatment outcomes for MDR-TB in migrant populations and factors impacting on adherence, TB control strategies are increasingly targeting migrant groups and there is an emphasis on improving the detection and treatment of TB and MDR-TB in migrants. A robust assessment of adherence to MDR-TB treatment regimens is, therefore, necessary to assess progress against treatment goals and to inform policy and practice.

## Methods

The aim of this systematic review and meta-analysis is, therefore, to identify and synthesise data on MDR-TB treatment adherence in migrant populations.

### Protocol and registration

This research was carried out in line with the Preferred Reporting Items for Systematic Reviews and Meta-Analyses (PRISMA) [29], and registered with PROSPERO (42017070756).

### Search strategy and inclusion and exclusion criteria

We included peer-reviewed papers reporting primary data on MDR-TB treatment adherence in migrants (foreign-born and receiving treatment outside their country of birth). Those studies reporting stipulated WHO outcomes for individuals receiving MDR-TB treatment that could be used as proxies for treatment adherence (cured, treatment completed, treatment failure, lost to follow-up or not evaluated) were included [30, 31], as well as studies reporting other outcomes in line with the WHO outcome measures (still on treatment, unsuccessful outcome or successful outcome) [32]. These treatment outcomes were included to comprehensively identify and examine available data on treatment adherence in migrants. The use of treatment outcomes as a proxy for treatment adherence enabled the most comprehensive examination of available data on treatment adherence among migrants, and is often used in population-based assessments. However, sensitivity analyses were also conducted to explore further treatment adherence specifically.

Studies were not excluded based on publication date or language, and non-English papers were translated prior to full-text screening. There were also no restrictions on whether studies considered paediatric or adult populations, or the type of study design. Studies defining migrant status according to ethnic or ancestral background but not country of birth were excluded, as were papers where primary data were not reported (e.g. comments, editorials, letters and reviews).

We searched the databases Embase (1947 to 22 May 2017), Global Health (1973 to 24 May 2017), MEDLINE (1946 to 22 May 2017) and PubMed (1993 to 22 May 2017) using a Boolean search strategy with keywords and relevant medical subheadings (MeSH) pertaining to four main themes: migrants, adherence, tuberculosis and drug resistance (including MDR-TB). The terms used were identified by consulting the literature, previous systematic reviews [20, 33] and experts in these areas. The search strategy is available in the Appendix.

Additional relevant papers were also identified through hand searching the bibliographies of publications included after full-text screening, as well as related information sources, including: the Global Fund, Public Health England, WHO and the International Union Against Tuberculosis and Lung Disease. Experts in the field were also consulted to identify additional relevant papers.

### Screening and data management

Two reviewers duplicated the title and abstract screening and full-text screening (LBN and KR), which was carried out using the web-based application Rayyan [34].

Data extraction and quality assessment for all papers included were also conducted by two reviewers (LBN and KR). Using a piloted data extraction form, summary data were extracted on study design, dates, location, patient characteristics, type of treatment, and migrant and non-migrant treatment outcomes in line with WHO outcome measures. Quality assessment was carried out using established appraisal tools. All case series were assessed using the Case Series Critical Appraisal Tool of the Joanna Briggs Institute [35]. Case–control studies were assessed using the Critical Appraisal Skills Program (CASP) appraisal tool [36]. Cohort studies were assessed using the CASP cohort checklist [37]. Using these tools, papers were given a quality score. For case-series and cohort tools, scores were calculated as a total out of the maximum number of applicable questions.

### Data analysis

Statistical analysis was carried out using Stata 13 [38]. The commands *metaprop* and *metan* were used to calculate pooled prevalence and pooled risk ratios (RRs), respectively, with corresponding 95% confidence intervals (CIs) [39, 40]. Heterogeneity between studies was examined using the *I*^2^ statistic. Due to the heterogeneity of the included studies, analyses utilised random-effects models [40].

We estimated the pooled proportion of migrants who were adherent and not adherent to treatment. We carried out analyses for both of these categories, as neither capture individuals who have died. We defined adherent individuals as those reported as cured, having completed treatment or with a successful outcome, utilising these treatment outcomes as indicators of treatment adherence to examine all available evidence comprehensively. Individuals considered adherent have, therefore, had confirmation of MDR-TB cure or have satisfactorily completed a full course of treatment. Successful outcomes cover both of these categories. Those still on treatment were excluded from the adherent variable as data were not available on treatment outcome. However, sensitivity analyses were conducted to examine the effect of these patients on pooled adherence rates across the studies if they were adherent or non-adherent.

We defined non-adherent individuals as those reported in the literature within categories: lost to follow-up, treatment default (discontinuation of treatment), treatment failure (which can often be attributed to issues in adherence) and unsuccessful outcome. Patients who transferred out of treatment were excluded from the non-adherent group as data on outcome were not known. A sensitivity analysis was conducted excluding treatment failure, as this outcome may be due to factors other than non-adherence.

We also carried out meta-analyses comparing rates of adherence and non-adherence between migrants and non-migrants across the included studies. These meta-analyses excluded studies in which no events occurred in either the migrant or non-migrant arms (both-armed zero-event studies [41, 42]), although these data are captured in the pooled proportions enabling comparison between migrants and non-migrants.

## Results

### Screening results

**Fig. 1:**
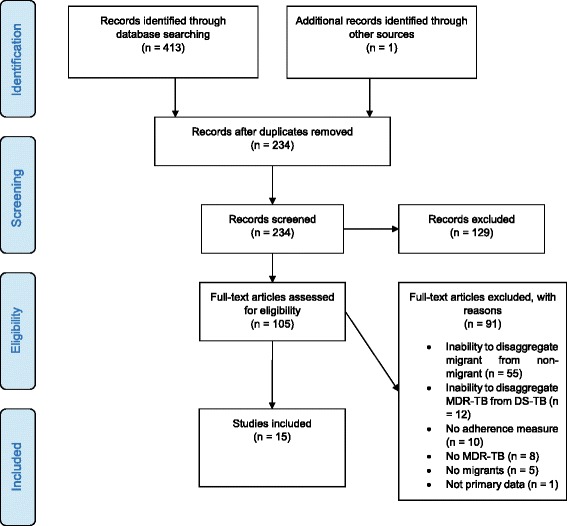
PRISMA flow diagram of study selection process [29]. It includes reasons for exclusion at the full-text screening stage. DS-TB drug-sensitive tuberculosis, MDR-TB multidrug-resistant tuberculosis

Database searches yielded 413 publications, with 234 publications carried forward for title and abstract screening after the removal of duplicates. Of those, 129 citations were excluded. The full texts of 105 publications were screened, including one citation identified through bibliography screening, hand searching of relevant sites, and recommendations from experts. Ninety one records were excluded during full-text screening, and the reasons for their exclusion recorded (Fig. 1). Altogether, 15 publications met the inclusion criteria and were included in the review and meta-analysis (Fig. 1).

### Study characteristics

The publications included in the analyses comprised seven cohort studies, six case series and two case–control studies, containing data from 1986 to 2014. All studies labelled as case series were described as such in the relevant papers, and are inclusive of all cases during the relevant study period for each data set. Studies were conducted in 11 countries: Canada (2) [43, 44], Australia (2) [32, 45], Czech Republic (1) [46], Finland (1) [47], France (1) [48], Germany (1) [49], Iran (1) [50], Italy (3) [51–53], New Zealand (1) [54], Switzerland (1) [55] and USA (1) [56] (Table 1). A number of studies indicated that treatment was provided at specialist TB centres or centres that otherwise specialised in infectious and pulmonary diseases [43, 46, 50, 52, 55], whilst the remaining studies either did not indicate the institutions providing treatment or were based on national-level data. Data were not consistently reported on migrant characteristics, including the reason for migration, time since migration or country of origin. Of the included studies reporting migrant status, it was typically stated only whether a patient was foreign-born, whilst two studies have a clearly identifiable population of cross-border and binational individuals who are largely migrating for treatment as opposed to permanent settlement [32, 56].

**Table 1:** Characteristics of studies included

Citation	Study type	Location	Years with data	Migrant (*N*)	Non-migrant (*N*)	Study quality
Avendano, 2000 [43]	Cohort	Canada	1986–1999	38	2	9/9
Bartu et al., 2010 [46]	Case–control	Czech Republic	2001–2009	17	33	7/9
Blaas et al., 2008 [49]	Case series	Germany	1997–2006	4	nr	9/9
Cameron & Harrison, 1997 [54]	Case series	New Zealand	1988–1995	7	1	7/9
Donnan et al., 2017 [32]	Case series	Australia	2005–2014	14	nr	9/10
Ferrara et al., 2005 [51]	Cohort	Italy	1995–1999	39	88	9/9
Ferrer et al., 2010 [56]	Cohort	USA	1994–2007	48	nr	9/9
Flament-Saillour et al., 1999 [48]	Case–control	France	1994–1996	32	19	8/9
Judge et al., 2016 [45]	Case series	Australia	2004–2013	6	nr	7/9
Kherad et al., 2009 [55]	Cohort	Switzerland	1999–2003	5	nr	11/11
Manfredi et al., 2009 [52]	Cohort	Italy	2006–2008	2	nr	7/9
Masjedi et al., 2008 [50]	Cohort	Iran	2002–2006	28	23	9/9
Mignone et al., 2014 [53]	Case series	Italy	2006–2010	1	1	8/9
Minion et al., 2013 [44]	Case series	Canada	1997–2008	3	2	10/10
Vasankari et al., 2012 [47]	Cohort	Finland	1994–2005	14	5	9/9

*nr* not reported

A total of 258 migrants with MDR-TB were included in the studies. For nine studies, we were able to disaggregate data for 174 non-migrants with MDR-TB or extensively drug-resistant tuberculosis [43, 44, 46–48, 50, 51, 53, 54].

The quality of the included studies was high, with the number of criteria met ranging from 7/9 to 11/11 for cohort studies, 7/9 to 10/10 for case series, and 7/9 [46] and 8/9 [48] for the two case–control studies included. Studies were not excluded based on study quality. Quality scores for each study are reported in Table 1.

### Comparable rates of MDR-TB treatment adherence among migrants and non-migrants

**Fig. 2:**
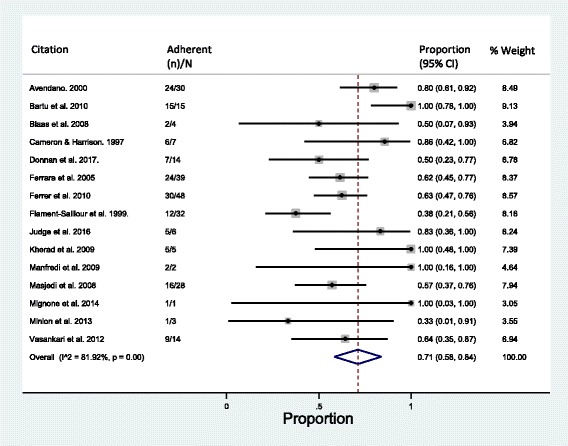
Pooled proportion of migrants adherent to MDR-TB treatment regimens. Treatment completion and cure are considered adherent. Individuals on treatment are excluded. CI confidence interval, MDR-TB multidrug-resistant tuberculosis

MDR-TB treatment adherence among the 248 migrants across the included studies was 71% (95% CI = 58–84%; *I*^2^ = 82%) after excluding individuals still on treatment (*n* = 10) (Fig. 2). Among those who were considered adherent, 76 were reported as cured, 36 as having completed treatment, with a further 47 reported as having successful outcomes. Sensitivity analyses in which migrants still on treatment were reintroduced gave a rate ranging from 66% to 72%, depending on whether the individuals were assumed to be non-adherent or adherent.

**Fig. 3:**
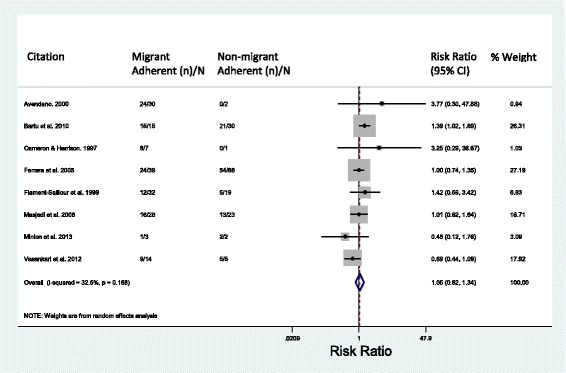
Meta-analysis of adherence in migrant vs. non-migrant populations. Treatment completion and cure are considered adherent. CI confidence interval

A meta-analysis was conducted utilising the nine studies for which the outcomes of 179 migrants and 171 non-migrants were disaggregated, enabling a comparison of MDR-TB treatment adherence in these two groups (Fig. 3). Migrant adherence to MDR-TB treatment was found to be comparable to non-migrant adherence (RR = 1.05, 95% CI = 0.82–1.34; *I*^2^ = 32.5%).

### Rates of non-adherence to MDR-TB treatment similar in migrants compared to non-migrants

**Fig. 4:**
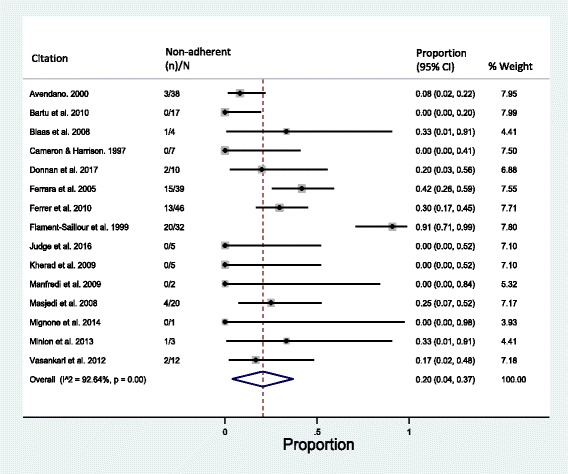
Pooled proportion of migrants not adherent to MDR-TB treatment. Treatment failure, discontinuation of treatment, loss to follow-up and unsuccessful outcomes are considered non-adherent. CI confidence interval, MDR-TB multidrug-resistant tuberculosis

Complementary analyses examining rates of non-adherence in migrants were also conducted, as patients who have died are not captured by either measure. Among the 248 migrants with reported MDR-TB treatment adherence outcomes, 61 were considered non-adherent while 17 individuals transferred out of treatment and were excluded. The estimated rate of non-adherence to MDR-TB treatment in migrants was 20% (95% CI = 4–37%; *I*^2^ = 67.32%) (Fig. 4). This rate included 17 individuals lost to follow-up, 23 who discontinued treatment and 21 individuals with reported treatment failure. However, when treatment failure was excluded, as this may be due to other factors besides non-adherence (and risks stigmatising patients through the attribution of treatment failure to poor adherence), the rate of non-adherence decreased to 11% (95% CI = 4–19%; *I*^2^ = 92.64%). In non-migrants, the rate of non-adherence when excluding treatment failure was 3% (95% CI = 0–8%; *I*^2^ = 9.92%).

**Fig. 5:**
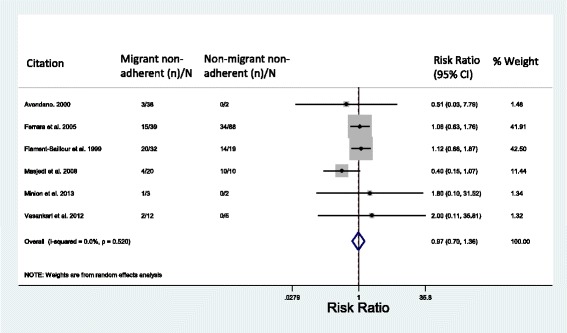
Meta-analysis of non-adherence to MDR-TB treatment regimens in migrant vs. non-migrant populations. Treatment failure, discontinuation of treatment, loss to follow-up and unsuccessful outcomes are considered non-adherent. CI confidence interval, MDR-TB multidrug-resistant tuberculosis

Rates of non-adherence to MDR-TB treatment were compared between migrants and non-migrants in six studies for which data were available (Fig. 5), and were found to be comparable in migrant and non-migrant populations (RR = 0.97, 95% CI = 0.70–1.36; *I*^2^ = 0%).

## Discussion

Adherence rates among migrants on treatment for MDR-TB were estimated to be 71% (95% CI = 58–84%), approaching global treatment adherence targets [12], and exceeding previous reports estimating adherence rates in the general population as 49–63.5% [57, 58]. This raises questions about whether more ambitious targets should be set for MDR-TB treatment adherence globally, and suggests that adherence may be dependent on social risk factors and treatment context, not only migrant status.

Rates of MDR-TB treatment adherence and non-adherence were also found to be comparable between migrants and non-migrants (RR = 1.05, 95% CI = 0.82–1.34 and RR = 0.97, 95% CI = 0.70–1.36, respectively). These findings challenge previous assumptions that adherence to MDR-TB treatment is likely to be worse in migrants [1, 21, 59, 60].

Social risk factors, for example social deprivation, vulnerable housing, mental health concerns or other comorbidities, have been shown to present barriers to care in migrant populations, and may contribute to poorer treatment adherence [61–64]. Being a highly mobile population with poor access or entitlement to high-quality health care may also present significant barriers to adhering to treatment, as might language or health literacy barriers. The legal complexities of a migrant’s status can also lead to non-adherence, although at times it is involuntary. For example, the right to stay within a country whilst receiving treatment is a facilitator of greater adherence [62]. In some contexts, it may be that migrants may perceive that their adherence to treatment (and cure) may impact on their status (e.g. leave to remain) in host countries. However, there is also evidence to suggest that insecure migrant status is associated with non-completion of treatment, with migrants potentially hiding or absconding from treatment centres for fear of deportation [26, 62, 65]. There are even cases of illegal and undocumented migrants with TB being deported, interrupting treatment and likely facilitating the spread of resistance [66]. Practices such as the current sharing of patient data with the Home Office for immigration enforcement purposes in the UK may contribute to non-adherence, as patients are concerned that contact with health services may result in their deportation [67].

The health service context within which MDR-TB is diagnosed and treated may also be a significant determinant of adherence, and may mediate the effects that social and cultural barriers may have (particularly in the context of more intensive treatment programmes or hospitalisation) [24, 61, 68]. The findings may also be driven by an emphasis on individualised treatment regimens [57], with evidence that adherence was improved among patients in specialist institutions (e.g. centres specialising in TB or pulmonary diseases), which may be better equipped to support treatment adherence [27, 46, 48], though it was not feasible given the available data to conduct a meta-analysis on this. The overrepresentation of high-income low-TB-incidence countries across the studies, which may have health infrastructures that are better equipped to support treatment adherence in MDR-TB patients and may also have targeted care or support for migrant service users, may also have contributed to the treatment adherence rates seen [69].

Across the included studies, it was also suggested that adherence rates have increased in recent years, which is likely to reflect an improvement in the provision of MDR-TB treatment and increased availability of specialist TB services [70, 71]. Whilst other factors such as increasing TB programme visibility, provision of information, patient-centred interactions with health-care providers and approaches to minimising or managing treatment side effects are also likely to impact on treatment outcomes [72], data were not available to allow a comprehensive examination of these factors.

The comparable rates of adherence (and non-adherence) between migrants and non-migrants are in contrast with assumptions that adherence to TB treatment is likely to be worse in migrants [1, 21, 59, 60], and highlight the importance of ensuring that MDR-TB treatment pathways have the capacity to support patients experiencing risk factors for poor adherence, regardless of their migrant status. Furthermore, it is essential that patient groups are not marginalised or scapegoated because of their migrant status, but rather the focus of adherence-improvement strategies should remain on addressing key barriers to adherence.

This research represents the first systematic and comprehensive examination of adherence to MDR-TB treatment in migrant populations, to our knowledge. It identifies the need for a stronger evidence base in light of the lack of data collected on migrant status in TB and MDR-TB patients, though this group is a key focus of TB strategies in high-income low-TB-incidence countries given the increased burden of TB and MDR-TB in migrant populations in low-incidence countries [1, 2, 12], for example the Collaborative TB Strategy for England [73]. This should include a comprehensive examination of available evidence on adherence to drug-sensitive TB to complement this synthesis of data on MDR-TB, a key area for further investigation given the limited reporting of treatment completion by migrant status and the impact that poor adherence in treatment for drug-sensitive TB may have on the emergence of MDR-TB. Whilst the review illuminated some factors that may contribute to MDR-TB treatment adherence (or non-adherence), there remains a need for further research to improve understandings of drivers of MDR-TB treatment adherence and non-adherence within migrant populations, and to delineate where these mechanisms differ from non-migrant populations. Such research, including both quantitative and qualitative research, is needed to tailor interventions better to support treatment adherence in underserved groups and will require robust and consistent data collection around social risk factors and migrant status in MDR-TB patients.

Though this review addresses a significant gap in knowledge on MDR-TB treatment adherence in migrants, the research highlights a number of important limitations in the evidence base that should be taken into account when considering the results. First, the findings should be considered in the context of the use of treatment outcomes as a proxy for adherence, which enabled us to examine the available data comprehensively and highlights the key implications for clinical pathways.

The review highlighted the significant lack of research in which data are disaggregated in such a way that makes specific research into treatment adherence in MDR-TB and different risk groups feasible. This significantly limited the number of papers included in the review, with the majority of exclusions at full-text screening being attributed to an inability to disaggregate migrant status or drug susceptibility status in relation to treatment adherence. An additional factor limiting the inclusion of papers in this review is the inconsistent reporting of migrant status in the evidence base or frequent use of ethnicity as a proxy for migration. Whilst the review sought to identify peer-reviewed primary research reporting data on migrant groups comprehensively, papers that have not included routinely utilised migrant terms (see the Appendix) may not, thus, be identified in migrant health searches, such as that conducted here.

The total number of studies included in the analysis highlights the insufficient research on the relationship between migration and treatment adherence in MDR-TB. The lack of evidence in this area is concerning in light of the growing emphasis in both policy and practice in high-income countries on targeting migrants in the detection and treatment of MDR-TB [73–75].

Data across the included studies were also heterogeneous, reflecting the diverse migrant populations and settings represented. Whilst the analyses utilised random-effects models, it is also useful to interpret the findings in light of the differences across studies. A further limitation of the available data is the small sample sizes across studies, which are partly attributed to the low incidence of MDR-TB, high rates of loss to follow-up during the lengthy MDR-TB treatment pathway (and, thus, the small numbers of patients for whom treatment completion data are available), and our particular interest in migrants as a patient population. This only further highlights the need to strengthen the available evidence base in this area. There is also a risk of publication bias, as clinical data on MDR-TB treatment are not systematically analysed or published. Furthermore, the available data may reflect settings in which migrants are a specific interest group (e.g. due to being overrepresented), leading to decisions to record or stratify data based on migrant status, which was an inclusion criterion for this review.

## Conclusions

Adherence to MDR-TB treatment is critical both in reducing poorer and costlier individual health outcomes and in preventing the transmission of MDR-TB. Despite the comparable rates of adherence in migrants and non-migrants, there are still a concerning number of individuals failing to complete MDR-TB treatment, enabling the spread of MDR-TB. This, therefore, calls into question whether MDR-TB treatment targets are ambitious enough and highlights the paramount importance of increasing adherence in all patient groups, including migrants, with an emphasis on tailoring care based on social risk factors for poor adherence in addition to migrant status.

## Appendix

Listed are representative search terms used in database searches (Embase).

Brackets () denote MeSH terms highlighted by the database.

1. Migrant* OR (migrant) OR (migrant worker) OR migrat* OR (migration) OR refugee* OR (refugee) OR asylum seeker* OR (asylum seeker) OR foreigner* OR (foreign worker) OR foreign born OR non-native* OR Immigra* OR (immigration) OR (immigrant) OR emigra* OR (emigrant) OR (Emigrants) OR (emigration) OR oversea* OR foreign student* OR (foreign student) OR International student* OR traffick*
2. Adher* OR complian* OR default OR concordan* or treatment outcome* OR (treatment outcome) OR non adher* OR (patient compliance) OR non-complian*
3. tuberculosis OR (TB) OR (drug resistant tuberculosis) OR (Tuberculosis) OR (Mycobacterium tuberculosis) OR (extensively drug resistant tuberculosis) OR (multidrug resistant tuberculosis)
4. MDR* OR (drug resistance) OR (multidrug resistant tuberculosis) OR (multidrug resistance) OR multidrug resist* OR multi-drug resist* OR multidrug-resist* OR XDR* OR (extensively drug resistant tuberculosis) OR extensively drug resistant) OR TDR* OR (Totally drug resistant)
5. 1 AND 2 AND 3 AND 4

## Abbreviations

CASP: Critical Appraisal Skills Program
CI: Confidence interval
DS-TB: Drug-sensitive tuberculosis
MDR-TB: Multidrug-resistant tuberculosis
MeSH: Medical subheadings
PRISMA: Preferred Reporting Items for Systematic Reviews and Meta-Analyses
RR: Risk ratio
TB: Tuberculosis
WHO: World Health Organization
